# Time-Resolved Effect of Interferon-Alpha 2a on Activities of Nuclear Factor Kappa B, Pregnane X Receptor and on Drug Disposition Genes

**DOI:** 10.3390/pharmaceutics13060808

**Published:** 2021-05-28

**Authors:** Dirk Theile, Lelia Wagner, Cindy Bay, Walter Emil Haefeli, Johanna Weiss

**Affiliations:** Department of Clinical Pharmacology and Pharmacoepidemiology, Heidelberg University Hospital, Im Neuenheimer Feld 410, 69120 Heidelberg, Germany; dirk.theile@med.uni-heidelberg.de (D.T.); lelia.wagner@uni-heidelberg.de (L.W.); cindy.bay@med.uni-heidelberg.de (C.B.); walter.emil.haefeli@med.uni-heidelberg.de (W.E.H.)

**Keywords:** interferon-alpha, pregnane X receptor, nuclear factor kappa B, cytochrome P-450 3A4, pharmacokinetic drug–drug interactions

## Abstract

Interferon-alpha (IFN-α) is suggested to cause pharmacokinetic drug interactions by lowering expression of drug disposition genes through affecting the activities of nuclear factor kappa B (NF-ĸB) and pregnane X receptor (PXR). The time-resolved impact of IFN-α 2a (1000 U/mL; 5000 U/mL; 2 h to 30 h) on the activities of NF-ĸB and PXR and mRNA expression (5000 U/mL; 24 h, 48 h) of selected drug disposition genes and on cytochrome P450 (CYP3A4) activity in LS180 cells (5000 U/mL; 24 h, 48 h) was evaluated using luciferase-based reporter gene assays, reverse transcription polymerase chain reaction, and luminescence-based CYP3A4 activity assays. The cross-talk between NF-ĸB activation and PXR suppression was evaluated by NF-ĸB blockage (10 µM parthenolide). IFN-α 2a initially (2 h, 6 h) enhanced NF-ĸB activity 2-fold and suppressed PXR activity by 30%. mRNA of *CYP3A4* was halved, whereas *UGT1A1* was increased (1.35-fold) after 24 h. After 48 h, *ABCB1* expression was increased (1.76-fold). CYP3A4 activity remained unchanged after 24 h, but was enhanced after 48 h (1.35-fold). IFN-α 2a demonstrated short-term suppressive effects on PXR activity and *CYP3A4* mRNA expression, likely mediated by activated NF-ĸB. Longer exposure enhanced CYP3A4 activity. Clinical trials should evaluate the relevance by investigating the temporal effects of IFN-α on CYP3A4 using a sensitive marker substrate.

## 1. Introduction

Interferons are important signaling molecules of the innate immune system. After pathogen-mediated activation of nuclear factor kappa B (NF-ĸB), type 1 interferons (e.g., interferon-alpha, IFN-α) and other proinflammatory cytokines (e.g., interleukin-6; tumor necrosis factor-alpha, TNF-α; etc.) are released to initiate anti-infectious responses [1]. Given the additional antiproliferative (antineoplastic) and immune response-regulating effects of IFN-α, this compound attracted scientific and clinical attention for more than five decades, and was developed as a therapeutic drug [1] against several cancers (e.g., cutaneous T-cell lymphoma, chronic myeloid leukemia, melanoma, and renal cell carcinoma) and virus hepatitis B and C [2,3]. IFN-α administration can induce an inflammatory phenotype (causing fever, myalgia, and headache) [4,5], which was associated with diminished clearance of small molecule drugs [6,7]. In humans, IFN-α administration was shown to lower drug clearance [8,9], raising the possibility of pharmacokinetic drug–drug interactions with coadministered compounds. However, the actual evidence for this assumption remains uncertain. While the impact of long-term IFN-α administration on cytochrome P450 isozyme 1A2 (CYP1A2) expression or pharmacokinetics of its prototypical substrate theophylline was convincingly shown in vitro [10], in healthy volunteers [11], and also in patients with viral hepatitis [12,13], the effect on the most prominent drug-metabolizing enzyme CYP3A4 is contradicting. Some clinical trials suggested that IFN-α suppresses CYP3A4 activity [14,15], whereas others found no change [16] or even enhanced CYP3A4 activity [17]. The interpretation is further complicated by the fact that these studies were made after different durations of IFN-α exposure (short-term: [12,16]; long-term: [11,13,16]). CYP3A4 expression and activity is tightly regulated by the nuclear pregnane X receptor (PXR) [18], so investigation of the time-resolved influence of IFN-α on this transcription factor should provide insight into the regulation of CYP3A4 and other drug disposition genes. To date, a previous experimental study suggested that cytokines (e.g., interleukin-6) can indeed antagonize PXR activity and subsequently diminish PXR target gene expression by activating NF-ĸB [19]. Whether this mechanism also applies to IFN-α is yet unclear.

In summary, previous studies suggest that IFN-α activates NF-ĸB, leading to inhibition of PXR and subsequent downregulation of PXR target genes (e.g., *CYP3A4*). However, a functional confirmation of the NF-ĸB:PXR cross-talk and a precise time-resolved analysis of the IFN-α effect on NF-ĸB, PXR, target gene expression or activity, and the respective temporal relations were not yet described. We therefore investigated the dual effect of IFN-α on the activities of both NF-ĸB and PXR and examined the temporal effect of changes on genes from different classes of drug disposition: the drug-metabolizing enzymes *CYP3A4* and *CYP1A1*, the drug-conjugating enzyme UDP-glucuronosyltransferase 1A1 (*UGT1A1*) and two members of the ATP-binding cassette transporter family called *ABCB1* (encoding P-glycoprotein) and *ABCC2* (encoding multidrug resistance-associated protein 2). IFN-α 2a and IFN-α 2b have identical indications, efficacy, and safety profiles [20,21], and showed similar suppressive effects on drug-metabolizing enzymes in vitro [22,23]; thus, these two forms can be used interchangeably. In this experimental study, IFN-α 2a was used. 

## 2. Materials and Methods

### 2.1. Material

Dulbecco’s Modified Eagle’s Medium (DMEM), Hanks’ balanced salt solution, phosphate-buffered saline (PBS), and the GenElute Mammalian Total RNA Miniprep Kit were purchased from Sigma–Aldrich (Taufkirchen, Germany). Fetal calf serum (FCS) was obtained from Biochrom (Berlin, Germany). Glutamine and nonessential amino acids were from Life Technologies (Paisley, UK). Rifampicin, dimethyl sulfoxide (DMSO), and Triton^®^ X-100 were from AppliChem (Darmstadt, Germany). IFN-α 2a was purchased from Life Technologies (Thermo Fisher Scientific (Waltham, MA, USA)), while parthenolide was obtained from Abcam (Berlin, Germany), and poly I:C from Invivogen (Toulouse, France). The cytotoxicity detection kit (LDH) was obtained from Roche Applied Sciences (Mannheim, Germany). The QuantiTect^®^ Primer Assay for *UGT1A1* was obtained from Qiagen (Hilden, Germany), the RevertAid H Minus First Strand cDNA Synthesis Kit from Thermo Fisher Scientific (Waltham, MA, USA). The Absolute QPCR SYBR Green Mix was purchased from ABgene (Hamburg, Germany). Primers were synthesized by Eurofins MWG Operon (Ebersberg, Gemany). The P450-Glo CYP Screening Systems and the Dual-Glo Luciferase Assay System, the pGL4.21 vector, the pGL4.74 [hRluc/TK] renilla vector, the pNL3.2.NF-κB-RE [NlucP/NF-κB-RE/Hygro] vector, and FuGENE^®^ HD Transfection Reagent were obtained from Promega Corporation (Madison, WI, USA). The *NR1I2* (NM_003889) Human cDNATrueClone^®^ (pCMV6-XL4 vector containing the cDNA of the PXR gene *NR1I2*) was obtained from OriGene (Rockville, MD, USA).

### 2.2. Stock Solutions

DMSO was used to prepare the stock solutions of parthenolide (100 mM) and rifampicin (100 mM). The stock solution of poly I:C (1 mg/mL) was prepared in 0.9% sodium chloride solution, while IFN-α 2a (5 × 10^7^ U/mL) was kept in the original stock solution provided by the manufacturer. The stock solutions were aliquoted into 500 µL reaction tubes and frozen at −20 °C.

### 2.3. Cell Line

The human colon adenocarcinoma cell line LS180 is a standard model for the investigation of the regulation of drug disposition genes, including PXR-mediated induction, and was shown to be superior to other models, such as HepG2 cells [24,25,26,27]. It is available at ATCC (Manassas, VA, USA) and was cultured under standard cell culture conditions with DMEM supplemented with 10% FCS, 2 mM glutamine, 100 U/mL penicillin, 100 μg/mL streptomycin sulphate, and 0.1 mM nonessential amino acids.

### 2.4. Cytotoxicity and Proliferation Assays

To rule out cytotoxic off-target effects (short-term incubation) or antiproliferative effects (long-term incubation), all compounds were initially tested in quadruplets for cytotoxicity with the Cytotoxicity Detection Kit according to the manufacturer’s instructions. In addition, growth inhibition assays were used to determine the maximum concentrations of IFN-α 2a, rifampicin, or poly I:C that had no antiproliferative effects in LS180 cells after 48-h exposure. Proliferation of LS180 cells was quantified by crystal violet staining, as described previously [28]. Antiproliferative properties were evaluated in three independent experiments with n = 4 wells for each concentration (500–50,000 U/mL). IFN-α 2a had no antiproliferative effects and reporter gene assays or gene induction assays were performed with 1000 U/mL to 5000 U/mL, which are concentrations in the range of previous experiments [10,23]. Accordingly, noncytotoxic or non-antiproliferative concentrations of rifampicin (20 µM) and poly I:C (1 µg/mL) were used.

### 2.5. Reporter Gene Assays

The effect of IFN-α 2a on the activities of NF-ĸB and PXR in LS180 cells was tested by luciferase-based reporter gene assays after treatment with 1000 U/mL or 5000 U/mL. NF-ĸB activity was recorded by transfecting LS180 cells with the pNL3.2.NF-κB-RE [NlucP/NF-κB-RE/Hygro] vector and the pGL4.74 [hRluc/TK] renilla vector using lipid-based FuGENE^®^ HD Transfection Reagent. For PXR evaluation, cells were transfected with the PXR overexpression vector (pCMV6-XL4), the pGL4.21-3A4-Luc, and pGL4.74 [hRluc/TK] renilla vector [29]. Twenty-four hours after the transfections, the medium was changed to medium containing IFN-α 2a (1000 U/mL; 5000 U/mL) or the positive controls (poly I:C, 1 µg/mL; rifampicin, 20 µM) and incubated for 2 h, 6 h, 24 h, or 30 h. The firefly luciferase activities (either driven by activated NF-ĸB or PXR) were recorded with the Dual-Glo Luciferase Assay System according to the manufacturer’s instructions. Luminescence was measured in a Glomax 96 microplate luminometer (Promega Corporation, Madison, MI, USA). Drug-induced enhancements of nuclear receptor activity were calculated by dividing firefly luminescence (modulated by NF-ĸB or PXR, respectively) by renilla luminescence (constitutive expression; controlling for variable transfection efficiency) and normalizing to nuclear receptor activities of untreated cells at the respective time point (set to 1). The assays were conducted in three independent experiments for each time point, with each concentration tested in technical triplicates.

To scrutinize whether IFN-α 2a-mediated activation of NF-ĸB is causally linked to suppression of PXR, NF-ĸB activation was blocked by the noncytotoxic parthenolide (10 µM), a sesquiterpene lactone that prevents NF-ĸB activation by binding to p65 or IĸB kinases [30,31].

### 2.6. Impact of IFN-α 2a on the Expression of Drug Disposition Genes

The impact of IFN-α 2a on the mRNA expression of *CYP3A4*, *CYP1A1*, *UGT1A1*, *ABCB1,* and *ABCC2* was tested after 24-h or 48-h treatment of LS180 cells. Cells were seeded and incubated for 3 days up to 70% confluency and then exposed in quadruplets to culture medium containing IFN-α 2a (5000 U/mL) for 24 h or 48 h. Rifampicin (20 µM) served as a positive control and culture medium without any experimental drug as a negative control. Harvested cells were used for RNA extraction.

### 2.7. Quantification of mRNA Expression by Real-Time RT-PCR

RNA was isolated using the GenElute Mammalian Total RNA Miniprep Kit, and the RevertAid H Minus First Strand cDNA Synthesis Kit was used to synthesize cDNA according to the manufacturers’ instructions. mRNA expression levels were quantified by real-time RT-PCR with a LightCycler^®^ 480 (Roche Applied Science, Mannheim, Germany), as described previously [32]. PCR amplification was performed in 20 µL reaction volume that contains 1× LC480 SYBR Green I Master and 5 µL 1:10 diluted cDNA. Primer sequences and obtained amplification product sizes are shown in supplemental Table S1. For quantification of *UGT1A1*, a QuantiTect® Primer Assay was used. The most stable housekeeping gene for normalization was identified via geNorm software (version 3.4, Center for Medical Genetics, Ghent, Belgium) [33]. From a panel of eight housekeeping genes tested, the one that was most stably expressed under each experimental condition (drugs; exposure times) was used for normalization (*glucose-6-phosphate dehydrogenase*; *beta-2-microglobulin*; *glucuronidase-beta*, *hypoxanthine-guanine-phosphoribosyltransferase*). Data was analyzed by a calibrator-normalized relative quantification approach with efficiency correction using the LightCycler^®^ 480 software version 1.5 (Roche Applied Science, Mannheim, Germany). Results are expressed as the target/reference ratio divided by the target/reference ratio of the calibrator. This approach corrects for sample inhomogeneities and variance caused by detection. All samples were amplified in two technical replicates.

### 2.8. Impact of IFN-α 2a on CYP3A4 Metabolic Activity

To evaluate the effect of IFN-α 2a on CYP3A4 function in LS180 cells after 24-h or 48-h exposure to 5000 U/mL, the P450-Glo™ CYP3A4 Assay (Luciferin-IPA) was used according to the manufacturer’s instructions and previous publications [34]. In brief, after treatment for 24 h or 48 h, cells (seeded in 96-well plates) were washed once with PBS and exposed for 60 min at 37 °C to 3 μM luciferin-IPA. Subsequently, 25 μL of the medium of each well (containing the biotransformed luciferin) was transferred to a fresh 96-well luminometer plate. After adding 25 μL of the Luciferin Detection Reagent, samples were incubated for 20 min at room temperature. Luminescence was measured in a GloMax luminometer. Recorded values were corrected for background luminescence and normalized to the number of viable cells (assessed by crystal violet staining). Relative changes of CYP3A4 activity were calculated by dividing drug-treated wells by the mean of the negative control wells. Rifampicin (20 µM) served as a positive control. The assays were conducted in three independent experiments for each time point each in technical octuplets.

### 2.9. Statistical Analysis

mRNA expression data and time-resolved results of the reporter gene assays were analyzed with an unpaired, nonparametric, two-sided Mann–Whitney U-test. Data on the effects of parthenolide cotreatment with IFN-α 2a on NF-ĸB or PXR activities were analyzed with an unpaired, nonparametric two-sided Kruskal–Wallis test. The effects of parthenolide alone or together with rifampicin on PXR activity were evaluated by nonparametric Kruskal–Wallis test and Dunn’s multiple comparison test. The effect of IFN-α 2a on CYP3A4 metabolic activity was evaluated using a two-way ANOVA with Dunnett’s multiple comparison posthoc test. Changes were regarded significant if *p* ≤ 0.05.

## 3. Results

### 3.1. Impact of IFN-α 2a on NF-ĸB or PXR Activities over Time

**Figure 1:**
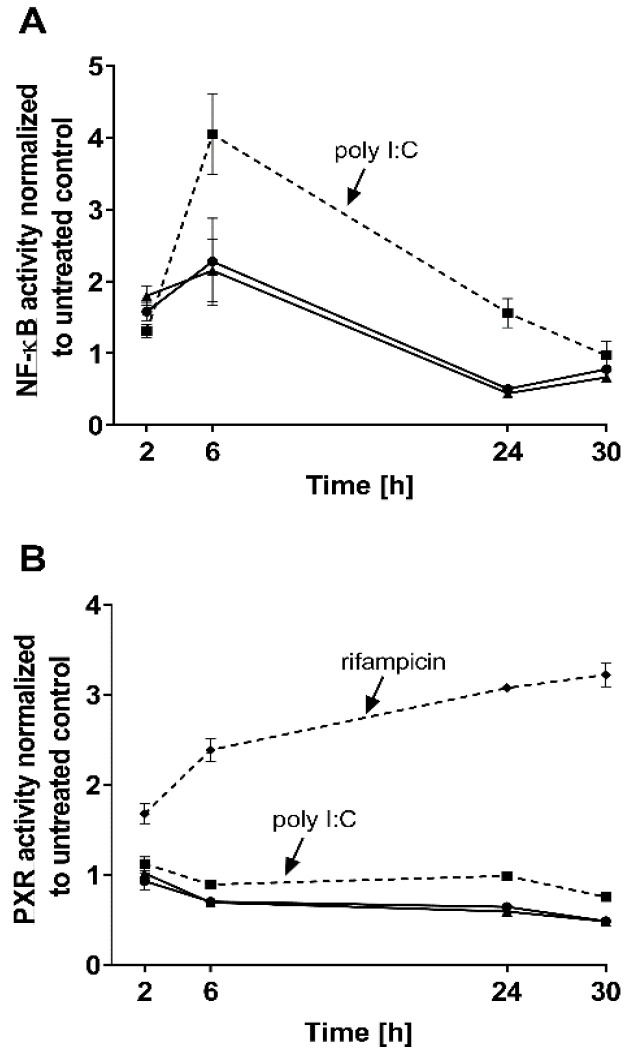
Impact of IFN-α 2a (1000 U/mL, black circles; 5000 U/mL, black triangles) on activity of NF-ĸB (**A**) and PXR (**B**) in LS180 cells, after exposure for 2 h, 6 h, 24 h, or 30 h. Luminescence values of drug-treated samples were normalized to luminescence of untreated control samples. Data shown are the mean ± S.E.M. of three independent experiments with technical triplicates for each time point. Poly I:C (1 µg/mL, black squares with dotted line) served as a positive control for NF-ĸB activation (**A**), whereas rifampicin (20 µM, black diamonds with dotted line) was used as a positive control for PXR activation (without IFN-α 2a) (**B**). Statistical significance (see text and Table 1 for details) was evaluated using an unpaired, nonparametric, two-sided Mann–Whitney U-test comparing drug-treated samples with control samples of respective time point. Control data sets (poly I:C and rifampicin) were published before [35].

**Figure 2:**
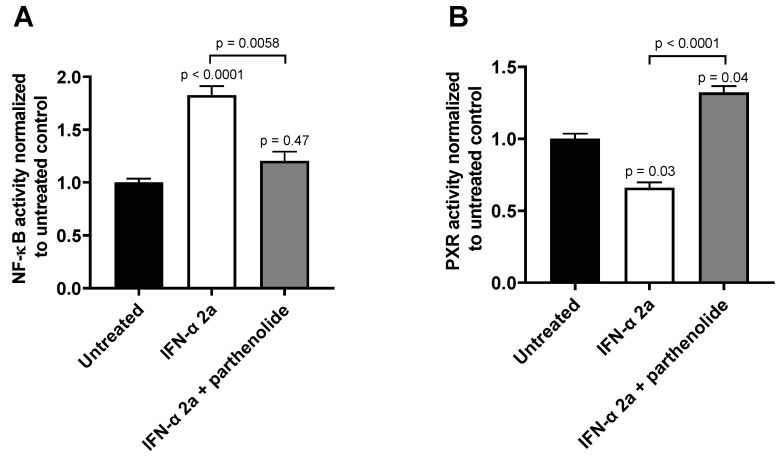
Effect of parthenolide (10 µM) on IFN-α 2a-mediated activation of NF-ĸB (**A**) or suppression of PXR (**B**) in LS180 cells after 6 h of exposure to 5000 U/mL of IFN-α 2a. Data shown are the mean ± S.E.M. of three independent experiments with technical quadruplets each. Statistical significance was evaluated using a nonparametric Kruskal–Wallis test and Dunn’s multiple comparison test. Single *p* values refer to comparison with untreated controls, whereas the *p* values above line show significance between treatments being connecting by this line. *p* values < 0.05 were considered significant.

The effects of both IFN-α 2a concentrations (1000 U/mL; 5000 U/mL) were biphasic; initially, INF-α 2a increased NF-ĸB activity with an observed peak at 6 h (2-fold increase compared with untreated control), whereas it suppressed NF-ĸB activity at 24 h (approximately 0.5-fold) and 30 h (approximately 0.7-fold) (as illustrated in Figure 1A, Table 1). Concurrently, PXR activities were significantly reduced after 6 h (0.7-fold), 24 h (0.6-fold), and 30 h (0.5-fold), compared to that of that of the untreated control (as illustrated in Figure 1B, Table 1). Poly I:C, as a positive control, clearly activated NF-ĸB at all time points (2 h: 1.3-fold, *p* < 0.0001; 6 h: 4.0-fold, *p* < 0.0001; 24 h: 1.55-fold, *p* = 0.0078) except after 30 h (*p* = 0.2224) (as illustrated in Figure 1A), and lowered PXR activity after 6 h (0.89-fold; *p* = 0.04) and 30 h (0.75-fold, *p* = 0.0005) (as illustrated in Figure 1B). The positive control for PXR activation (rifampicin, 20 µM) enhanced PXR at all time points (2 h: 1.67-fold, *p* = 0.0002; 6 h: 2.38-fold, *p* < 0.0001; 24 h: 3.0-fold, *p* < 0.0001; 30 h: 3.21-fold, *p* < 0.0001) (as illustrated in Figure 1B). These positive control data sets were published before [35]. To confirm the mechanistic link between NF-ĸB activation and subsequent PXR suppression, parthenolide (10 µM) was used together with IFN-α 2a (5000 U/mL) for 6 h, the time point of largest discordance between activities of NF-ĸB or PXR. Parthenolide diminished IFN-α 2a-mediated NF-ĸB activation (IFN-α 2a alone: 1.82-fold compared to that of untreated controls; IFN-α 2a + parthenolide: 1.2-fold compared to that of untreated control; *p* = 0.0058) (as illustrated in Figure 2A) and reversed PXR suppression (IFN-α 2a alone: 0.66-fold compared to that of untreated controls; IFN α 2a + parthenolide: 1.32-fold compared to that of that of untreated control; *p* < 0.0001) (as illustrated in Figure 2B). Because parthenolide cotreatment slightly over-compensated for the PXR suppression, control experiments were performed to exclude nonspecific effects of parthenolide. When used alone, parthenolide neither influenced the effect of the positive control drug for PXR activation rifampicin (rifampicin alone: 1.87-fold compared to that of untreated control; rifampicin + parthenolide: 2-fold compared to that of untreated control; *p* > 0.99) nor activated PXR (1.24-fold compared to that of untreated control; *p* = 0.96) (as illustrated in Supplemental Figure S1).

### 3.2. Impact of IFN-α 2a on mRNA Expression of Selected Drug Disposition Genes

**Figure 3:**
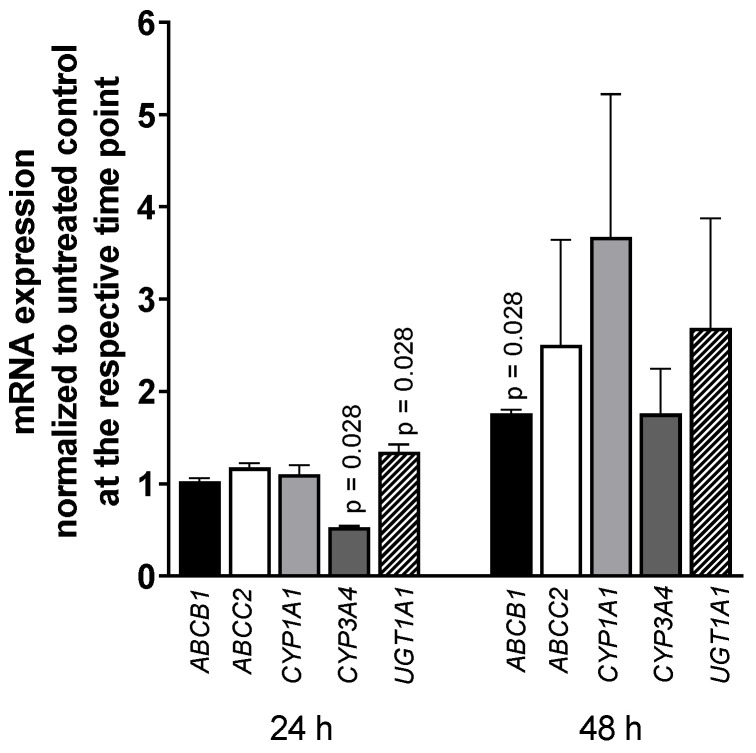
mRNA expression levels of selected drug disposition genes in LS180 cells after treatment with 5000 U/mL IFN-α 2a for 24 h or 48 h. mRNA expression levels of exposed samples were normalized to expression levels of untreated control samples. Data shown are the mean ± S.E.M. of four independent experiments with technical duplicates for each compound, gene, or time point, respectively. Significant differences between drug-treated samples and corresponding controls were evaluated by an unpaired, nonparametric, two-sided Mann–Whitney U-test. *p* values < 0.05 were considered significant.

Exposing LS180 cells to 5000 U/mL IFN-α 2a for 24 h or 48 h reduced the mRNA level of CYP3A4 (0.52-fold compared to that of untreated control; *p* = 0.028), whereas UGT1A1 was increased (1.35-fold; *p* = 0.028) after 24-h exposure as was ABCB1 after 48 h (1.76-fold; *p* = 0.028) (as illustrated in Figure 3). All other genes or time points did not reach statistical significance. The positive control rifampicin (20 µM) increased mRNA expression of ABCB1 (2-fold) after 24 h and 48 h (*p* = 0.0286) and CYP3A4 (5-fold, *p* = 0.0286) and UGT1A1 (2-fold, *p* = 0.0286) after 48-h exposure (data not shown; positive control data were published previously [35]).

### 3.3. Impact of IFN-α 2a on CYP3A4 Activity

**Figure 4:**
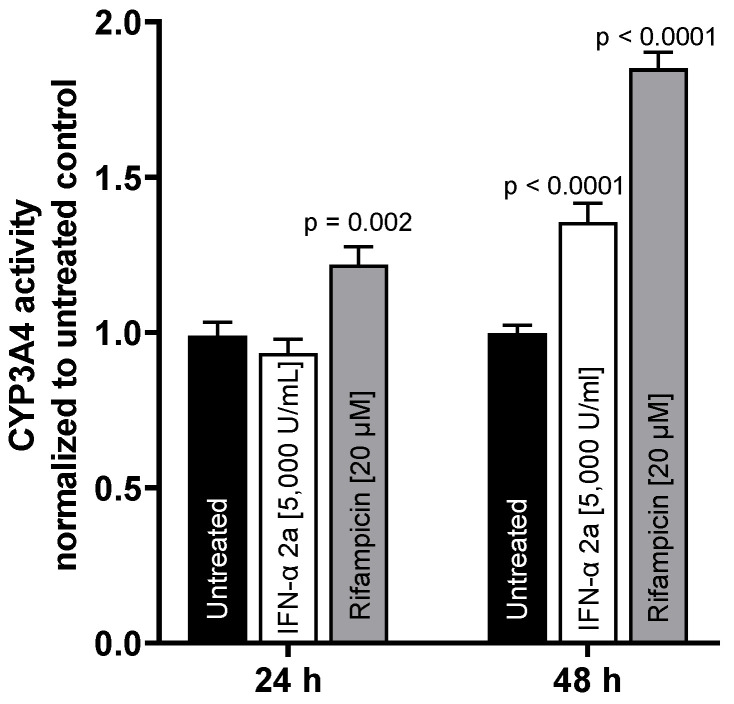
CYP3A4 metabolic activity in LS180 cells after exposure to 5000 U/mL IFN-α 2a (white bars) or 20 µM rifampicin (gray bars) for 24 h or 48 h, respectively. Luminescence values of drug-treated samples were normalized to luminescence of untreated control samples (black bars). Data shown are the mean ± S.E.M. of three independent experiments with 6–7 technical replicates each. Statistical significance was evaluated using a two-way ANOVA with Dunnetts’s multiple comparison test, comparing drug-treated samples to control samples. *p* values < 0.05 were considered significant.

To test whether mRNA expression translates into activity changes, CYP3A4 metabolic activity was evaluated. While there was no effect after 24 h, IFN-α 2a (5000 U/mL) enhanced CYP3A4 activity after 48 h (1.35-fold compared to that of untreated control; *p* < 0.0001). The positive control rifampicin (20 µM) enhanced CYP3A4 activity at both time points (24 h: 1.21-fold, *p* = 0.002; 48 h: 1.85-fold, *p* < 0.0001) (as illustrated in Figure 4).

## 4. Discussion

IFN-α is an endogenous cytokine and also an important antineoplastic and antiviral drug. However, drug interactions are not thoroughly investigated and its potential to alter the disposition of small molecule drugs is still debated. Therefore, we investigated the effects of IFN-α 2a on the expression of a selected set of drug disposition genes. Because NF-ĸB activation was suggested to be mechanistically involved in PXR inhibition and CYP suppression [19], we assessed the time-resolved activities of NF-ĸB and PXR and evaluated their functional cross-talk.

IFN-α 2a clearly activated NF-ĸB during the first 6 h and diminished it below baseline after 24 h or 30 h of continuous exposure (as illustrated in Figure 1A). Such a biphasic kinetic response with an observed activity peak within the first hours is typical for NF-ĸB activation by immunological stimuli [36,37,38]. Concurrently, IFN-α 2a-mediated activation of NF-ĸB was accompanied by suppression of PXR activity (as illustrated in Figure 1B). The involvement of NF-ĸB in the PXR response was further confirmed by showing that the NF-ĸB inhibitor parthenolide blunted IFN-α 2a-mediated NF-ĸB activation and concurrently prevented PXR suppression (as illustrated in Figure 2). Parthenolide in fact slightly overcompensated the PXR-suppressive effects of IFN-α 2a, but enhancement of basal PXR activity by strong NF-ĸB inhibition was already observed previously [19].

When IFN-α suppresses PXR activity, expression levels of PXR-regulated target genes should decline accordingly. Considering that the estimated half-life of *CYP3A4* mRNA in LS180 cells is approximately 12 h [39], mRNA expression levels were evaluated after 2 and 4 half-lives of continuous exposure (i.e., at quasi-steady-state). Whereas *CYP3A4* mRNA was reduced after 24 h, other PXR-regulated genes were unaffected or even became induced (*ABCB1* after 48 h; *UGT1A1* after 24 h) (as illustrated in Figure 3). In addition, CYP3A4 metabolic activity also was unchanged after 24 h and became induced after 48 h (as illustrated in Figure 4), reaching approximately half the effect size of rifampicin. Such apparent inconsistencies are difficult to explain but might be related to the diverse nature of protein complexes implicated in the regulation of distinct drug disposition genes [40] and its additional cross-talk with interferon signaling. IFN-α triggers the well-known Janus kinase (JAK)/signal transducer and activator of transcription (STAT) ‘immunological’ pathway initially [41], whereas the alternative pathway through the CAAAT/enhancer binding proteins (C/EBPs) were described responsible for rather long-term interferon effects [41,42], including their impact on CYP3A4 expression [43]. Of these proteins, C/EBP-α and C/EBP-β mediate CYP3A4 induction, whereas a truncated C/EBP-β protein downregulates CYP3A4 [44,45]. Most importantly, the time-dependent stoichiometric ratio and competition between these inducible C/EBPs proteins eventually determine the net effect [44,45]. Moreover, the initial cytokine-mediated downregulation of *CYP3A4* with subsequent rebound observed in this study was also documented [23,44].

Summarizing this data, it certainly seems coherent that IFN-α 2a initially suppresses PXR activity and mRNA expression of drug disposition genes (through NF-ĸB:PXR cross-talk), which acutely does not change expression or activity of the proteins given their respective protein half-lives. After longer exposure, IFN-α can enhance CYP3A4 at the functional level, consistent with data from patients with viral hepatitis chronically treated with IFN-α [17]. Translating in vitro findings to responses in humans is always difficult, but compared to that of the 85% increase by rifampicin (a clinically very strong perpetrator drug), the observed 35% increase of CYP3A4 activity by IFN-α is certainly remarkable, albeit the relevance of PXR for this induction is uncertain. Our findings suggest that alternative signaling pathways (e.g., C/EBPs proteins) are more likely to play a role in such IFN-α inductions than PXR, which is more likely responsible for induction by small molecules. For instance, when LS180 cells were treated with endothelin-1 receptor antagonists, phosphodiesterase 5 inhibitors [46], or rifampicin [24], reporter assay responses, mRNA levels, protein levels, and functional effects of PXR-regulated genes (e.g., P-glycoprotein, CYP3A4) correlated proportionately.

This study has some limitations. Although many proteins and signaling pathways are implicated in drug disposition, only PXR and a selection of genes or enzymes were evaluated, thus limiting generalization to other drug disposition genes and proteins. Moreover, like any other in vitro experiment, the data presented here unlikely reflects the diverse clinical situations influencing the net effect of IFN-α (viral hepatitis vs. tumor disease without hepatic impairment). Despite these weaknesses, the study also has some strengths. Because PXR is an important modulator of expression of drug metabolism genes, the preclinical investigations presented here were performed in a cellular standard model for PXR activity alterations, including genes from different classes of drug disposition (drug metabolism, drug transport, and drug conjugation), and used carefully selected proper positive controls. Finally, because CYP3A4 is the most important isozyme involved in drug metabolism, its enzymatic activity in LS180 cells was eventually monitored at a functional level (not only RNA level) after two different times of IFN-α 2a exposure. Finally, this study’s strength and novelty is the time-resolved description of IFN-α 2a effects on NF-ĸB and PXR and the experimental confirmation of their functional cross-talk.

In conclusion, this in vitro study confirms that IFN-α 2a initially suppresses activity and target gene expression of PXR (*CYP3A4*) by NF-ĸB activation. However, longer exposure to IFN-α can also have inducing effects, likely independent from PXR. Unless clinical trials thoroughly characterize IFN-α as a perpetrator drug or clearly describe the conditions modulating its impact, monitoring of drug levels or effects may be warranted when IFN-α is coadministered with drugs exhibiting narrow therapeutic indices.

## Figures and Tables

**Table 1:** Statistical evaluation of relative NF-ĸB and PXR activities after different exposure times of LS180 cells to two different IFN-α 2a concentrations.

Exposure Time	NF-ĸB Activity		PXR Activity	
	IFN-α 2a 1000 U/mL	IFN-α 2a 5000 U/mL	IFN-α 2a 1000 U/mL	IFN-α 2a 5000 U/mL
2 h	**<0.0001**	**<0.0001**	0.546	0.666
6 h	**0.0078**	**0.002**	**<0.0001**	**<0.0001**
24 h	**<0.0001**	**<0.0001**	**<0.0001**	**<0.0001**
30 h	**0.010**	**0.0003**	**<0.0001**	**<0.0001**

Shown are *p* values evaluated by a nonparametric, unpaired, two-sided Mann–Whitney U-test. Significant changes (*p* ≤ 0.05) are highlighted in bold.

## Data Availability

Data is contained within the article.

## Supplementary Materials

The following are available online at https://www.mdpi.com/article/10.3390/pharmaceutics13060808/s1. Figure S1: effect of parthenolide (10 µM) on PXR activity or on the rifampicin-mediated activation of PXR in LS180 cells after 6 h of exposure to 50 µM rifampicin; Table S1: primers used for qPCR.
