# Supplementary material for: Time-Resolved Effect of Interferon-Alpha 2a on Activities of Nuclear Factor Kappa B, Pregnane X Receptor and on Drug Disposition Genes
Source: Pharmaceutics. 2021 May 28;13(6):808. doi: 10.3390/pharmaceutics13060808 (PMC8229072; doi:10.3390/pharmaceutics13060808)
Supplement: Supplementary file 1 [file pharmaceutics-13-00808-s001.zip › pharmaceutics-1214341-SI.pdf]

pharmaceutics

MDPI

Supplementary Materials: Time-Resolved Effect of Interferon-Alpha 2a on Activities of Nuclear Factor Kappa B, Pregnane X Receptor, and on Drug Disposition Genes

Dirk Theile, Lelia Wagner, Cindy Bay, Walter Emil Haefeli and Johanna Weiss

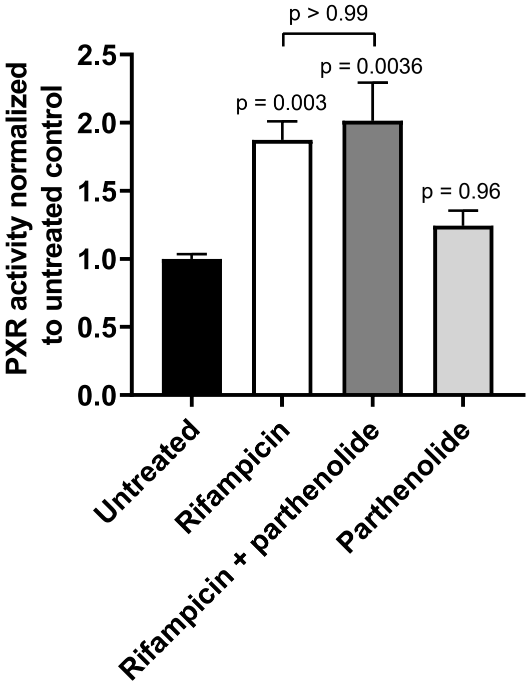

Figure S1: Effect of parthenolide (10 µM) on PXR activity or on the rifampicin-mediated activation of PXR in LS180 cells after 6 h of exposure to 50 µM rifampicin. Data shown is the mean ± S.E.M. of three independent experiments with technical quadruplets each. Statistical significance was evaluated using an ANOVA with non-parametric Kruskal-Wallis test and Dunn's multiple comparison test. Single P values refer to the comparison with untreated controls, whereas the p values above the line show the significance between the treatments being connecting by this line.

Table S1: Primers used for qPCR

Gene	Forward Primer 5′-3′	Reverse Primer 5′-3′	Product (bp)	Design
ABCB1	CCCATCATTGCAA-TAGCAGG	TGTTCAAACTTCTGCT CCTGA	158	[1]
ABCC2	ACAGAGGCTGGTGG-CAAC	ACCATTACCTTGTCAC-TGTCCATGA	226	[2]
CYP1A1	TCCGGGACATCACAG-ACAGC	ACCCTGGGGTTCATCA CCAA	183	[3]
CYP3A4	CAGCAAGAA-GAACAAGGACAA	GGTTGAA-GAAGTCCTCCTAAGC	153	[4]
UGT1A1	GAATCAACTGCCTTCA CCAAA	AC-CACAATTCCATGTTCT CCA	82	[5]

[1] Albermann, N.; Schmitz-Winnenthal, F.H.; Z'graggen, K.; Volk, C.; Hoffmann, M.M.; Haefeli, W.E.; Weiss, J. Expression of the Drug Transporters MDR1/ABCB1, and PXR in Peripheral Blood Mononuclear Cells and Their Relationship with the Expression in Intestine and Liver. *Biochem. Pharmacol.* **2005**, *70*, 949–958, doi:10.1016/j.bcp.2005.06.018.

[2] Pascolo, L.; Fernetti, C.; Pirulli, D.; Crovella, S.; Amoroso, A.; Tiribelli, C. Effects of maturation on RNA transcription and protein expression of four MRP genes in human placenta and in BeWo cells. *Biochem. Biophys. Res. Commun.* **2003**, *303*, 259-265. doi: 10.1016/s0006-291x(03)00327-9.

[3] Dvorak, Z.; Vrzal, R.; Henklova, P.; Jancova, P.; Anzenbacherova, E.; Maurel, P.; Svecova, L.; Pavek, P.; Ehrmann, J.; Havlik, R.; Bednar, P.; Lemr, K.; Ulrichova, J. JNK inhibitor SP600125 is a partial agonist of human aryl hydrocarbon receptor and induces CYP1A1 and CYP1A2 genes in primary human hepatocytes. *Biochem. Pharmacol.* **2008**, *75*, 580-588. doi: 10.1016/j.bcp.2007.09.013. Epub 2007 Sep 16.

[4] Cerveny, L.; Svecova, L.; Anzenbacherova, E.;Vrzal, R.;Staud, F.;Dvorak, Z.;Ulrichova, J.;Anzenbacher, P.;Pavek, P. Valproic acid induces CYP3A4 and MDR1 gene expression by activation of constitutive androstane receptor and pregnane X receptor pathways. *Drug Metab. Dispos.* **2007**, *35*, 1032-1041. doi: 10.1124/dmd.106.014456.

[5] Takeshita, A.; Igarashi-Migitaka, J.; Koibuchi, N.; Takeuchi, Y. Mitotane induces CYP3A4 expression via activation of the steroid and xenobiotic receptor. *J. Endocrinol.* **2013**, *216*, 297-305. doi: 10.1530/JOE-12-0297.
